# Assessment of Horizontal Semicircular Canal and Graviceptive Perception Before and After Cochlear Implantation

**DOI:** 10.3390/audiolres16050136

**Published:** 2026-09-17

**Authors:** Daniel Häussler, Dorotheea Cazan, Johannes Ludwig, Klaus Fraas, Angela Schell, Nicole Rotter, Lena Zaubitzer

**Affiliations:** 1 Medical Faculty Mannheim, Ruprecht-Karls-University of Heidelberg, 68167 Mannheim, Germany; 2 HNO Praxis Bad Schönborn, 76669 Bad Schonborn, Germany; 3 Department of Otorhinolaryngology Head and Neck Surgery, Helios Dr. Horst Schmidt Kliniken, 65199 Wiesbaden, Germany; 4 Department of Radiology and Nuclear Medicine, University Hospital Mannheim, 68167 Mannheim, Germany; 5 Department of Otorhinolaryngology Head and Neck Surgery, University Hospital Mannheim, 68167 Mannheim, Germany

**Keywords:** cochlear implantation, vestibular function, video head impulse test, subjective visual vertical, adults

## Abstract

**Background/Objectives:** Cochlear implantation (CI) effectively treats severe-to-profound sensorineural hearing loss, but its vestibular effects remain under investigation. This retrospective study assessed perioperative changes in horizontal canal function (video head impulse test, vHIT) and graviceptive perception (subjective visual vertical, SVV). **Methods:** Forty-three adults (mean age: 59 ± 15 years) implanted between May 2017 and July 2018 were assessed pre- and postoperatively. The prespecified primary analysis was the paired comparison of vHIT gain at 60 ms in the implanted ear, performed separately by implantation side; all other comparisons were exploratory. **Results:** Paired vHIT data were available for 36 of 43 participants. Gain in the implanted ear did not change after right-sided CI (n = 17; 1.52 ± 0.37 to 1.59 ± 0.63; change: +0.07, 95% CI: −0.41 to 0.56; t(16) = 0.32, *p* = 0.754), whereas it fell after left-sided CI (n = 19; median: 1.41 to 1.07; z = −3.02, *p* = 0.003); the two sides were not compared formally. Implanted and non-implanted ears did not differ postoperatively (n = 37; *p* = 0.116). Fourteen of thirty-six participants (38.9%) developed new ipsilateral catch-up saccades (exact McNemar *p* = 0.031 and 0.039). SVV deviation was 0.77 ± 0.47° and 0.92 ± 0.51° (*p* = 0.086). **Conclusions:** No significant change was detected after right-sided CI or in SVV; given the wide confidence interval and the small sample, this is not evidence of preserved function. The left-sided reduction, the newly developed ipsilateral catch-up saccades and the exploratory findings require confirmation in larger prospective studies.

## 1. Introduction

The auditory and vestibular systems are closely integrated sensory systems responsible for auditory perception, movement cognition, and balance [1]. Both systems share anatomical proximity in the os temporale and are innervated by the vestibulocochlear nerve [2]. Although CI does not directly target vestibular structures, the insertion of electrodes into the scala tympani can lead to vestibular impairments through mechanical trauma, perilymphatic leakage, inflammatory reactions, or electrical stimulation [3].

The vestibular system consists of the semicircular canals and the otolith organs (saccule and utricle), which detect angular and linear accelerations, respectively [4,5]. These structures are particularly vulnerable during CI surgery, given their close anatomical proximity to the cochlea. Studies have reported that vestibular impairment post-CI can manifest as dizziness, imbalance, or altered spatial orientation, with the saccule being the most frequently affected [6]. Histopathological studies confirm that CI can cause trauma to the vestibular end organs, potentially leading to temporary or permanent dysfunction [7].

Several pathophysiological mechanisms have been proposed to explain CI-induced vestibular dysfunction, including mechanical disruption of the labyrinth, endolymphatic hydrops, inflammatory responses, and direct electrical stimulation of vestibular structures [6,8]. It has been demonstrated that intraoperative perilymphatic leakage can lead to disturbances in the inner ear fluid homeostasis, potentially affecting both auditory and vestibular function [9].

Postoperative dizziness is a common complaint after CI, with incidence rates ranging from 9.3% to 17.4% in different studies [3]. Other studies, however, report that the incidence of vestibular lesions following CI ranges from 23% to 100% [10]. The systematic review and meta-analysis by Hänsel et al. which comprises 116 studies indicated that 17.4% of CI recipients experience new-onset dizziness, while 11.6% report a change in their dizziness symptoms [3]. Although some patients adapt through central compensation mechanisms, others experience prolonged vestibular deficits [11]. The systematic review by Hänsel et al. mentioned earlier demonstrated that vestibular impairments are frequently observed after CI, particularly when assessed using objective vestibular tests such as caloric testing, vHIT, and vestibular evoked myogenic potentials. However, the extent of dysfunction varies across vestibular end organs and testing paradigms [3]. Similarly, Ibrahim et al. highlighted that different vestibular assessment tools yield heterogeneous results, reflecting the receptor-specific nature of vestibular dysfunction and differences in sensitivity of the applied tests [9].

Given the crucial role of the vestibular system in maintaining balance and spatial orientation, understanding the extent of its impairment post-CI is essential. This study seeks to assess horizontal canal-related vestibulo-ocular function and graviceptive perception pre- and postoperatively using complementary vestibular tests, including the horizontal video head impulse test (vHIT) and subjective visual vertical (SVV) measurements. These tests provide valuable insights into horizontal semicircular canal (SCC) function and otolith-related graviceptive processing, respectively, allowing for assessment of horizontal SCC function and graviceptive pathway-related perceptual orientation after CI. The vHIT allows for rapid, non-invasive assessment of SCC function by measuring the vestibulo-ocular reflex (VOR), with sensitivity to high-frequency head movements [12]. In contrast, SVV reflects otolith-related graviceptive processing and central integration of vestibular signals, providing complementary information on spatial orientation [13].

Although the previous literature suggests that otolith organs, particularly the saccule, may be affected after CI, the present study focused on horizontal SCC function assessed by vHIT and otolith-related graviceptive perception assessed by SVV because these examinations were part of the standardized perioperative vestibular protocol at our institution and were consistently available for retrospective analysis. Because cervical vestibular evoked myogenic potentials were not available, no statement regarding saccular function can be derived from the present data; this study therefore addresses horizontal SCC function and graviceptive perception rather than vestibular function as a whole.

The primary aim of this study was to evaluate preoperative-to-postoperative changes in horizontal canal-related vestibulo-ocular function and graviceptive perception following CI in adults, with a primary focus on horizontal SCC function assessed by vHIT (gain at 60 ms and presence of catch-up saccades) and a secondary assessment of otolith-related graviceptive perception using subjective visual vertical (SVV).

Secondary aims were as follows: (i) to compare vestibular outcomes between the implanted and non-implanted ear, (ii) to explore differences between left- and right-sided implantation, and (iii) to explore associations between vestibular function, electrode insertion depth, and subjective dizziness.

## 2. Materials and Methods

This retrospective clinical study was conducted at our university clinic, the Department of Otorhinolaryngology, Head and Neck Surgery between May 2017 and July 2018. The study period reflects the period for which the required pre- and postoperative vestibular assessments were available for retrospective analysis. More recent patients were not included in the present cohort. The study included 43 patients who underwent CI surgery and agreed to participate after providing written informed consent. The written informed consent was obtained prospectively during 2017 and 2018. All surgeries were performed by two experienced right-handed surgeons. All implantations were performed by a round window approach. The inclusion criteria comprised adults with postlingual deafness undergoing their first CI in the study ear. Exclusion criteria included diagnosed Ménière’s disease, as well as other known vestibular disorders (e.g., vestibular neuritis, bilateral vestibulopathy), significant visual impairments affecting SVV assessment, cochlear malformations, prior CI revision surgeries, and neurological conditions potentially affecting vestibular function. Further patients who could not provide informed consent were not included. No a priori sample-size calculation was performed; the sample size was determined by the number of patients with paired pre- and postoperative vestibular data available during the study period. Because the cohort was operated on in 2017 and 2018, the results reflect the surgical technique, electrode designs, and perioperative management in use at that time; slim lateral-wall and modiolar arrays, along with hearing-preservation concepts, have since been more widely adopted [14]. The present data should therefore be regarded as an analysis of a historical clinical cohort rather than as a direct representation of current CI practice.

Preoperative audiometric testing confirmed profound sensorineural hearing loss in all participants. As part of the routine preoperative CI assessment at our institution, all patients underwent pure-tone and speech audiometry, free-field audiometry, tympanometry, acoustic reflex testing, and ABR/BERA. Vestibular testing routinely included vHIT, while VEMPs and caloric testing were performed depending on the individual clinical findings.

Preoperative CT scans of the mastoid bone and evaluation of the cochlea were conducted. All patients underwent a second CT scan postoperatively with radiological analysis of insertion depth of the implant. The study was approved by the local ethical board (2017-568N-MA).

### 2.1. Vestibular Testing

The available perioperative vestibular assessment was performed preoperatively and repeated postoperatively to evaluate potential changes in vestibular function. For the present study, the vestibular assessment consisted of the horizontal vHIT and the SVV. Caloric testing, cervical and ocular vestibular evoked myogenic potentials (cVEMPs, oVEMPs), and vertical-canal vHIT were not part of the standardized vestibular assessment during the study period and were therefore not available for retrospective evaluation. Accordingly, horizontal semicircular canal function was assessed using the horizontal vHIT, while otolith-related graviceptive processing was assessed using the SVV. The battery therefore comprised horizontal vHIT and SVV only and does not constitute a comprehensive vestibular assessment.

vHIT gain was determined as the instantaneous ratio of eye velocity to head velocity at 60 ms after impulse onset, as computed by the device software; regression-based gain was not used. The vHIT was performed using the EyeSee Cam™ (Interacoustics A/S, Middelfart, Denmark) at 220 Hz. After calibration (~1.5 m target distance), seated patients fixated a point while the examiner applied ≥15 unpredictable horizontal head impulses per side (5–20°, 150–200 ms, 100–200°/s). To minimize measurement artefacts, care was taken during testing to ensure proper goggle fixation, appropriate strap tension, standardized target distance, and repeated calibration prior to data acquisition. The same device, calibration protocol, and target distance were used for the preoperative and the postoperative examination.

Trials with artifacts (e.g., goggle displacement, eye closure, non-uniform movement) were repeated; invalid data were algorithmically excluded. No additional predefined quality-control criterion excluding physiologically implausible gain values was applied; all technically valid recordings were retained.

**Figure 1:**
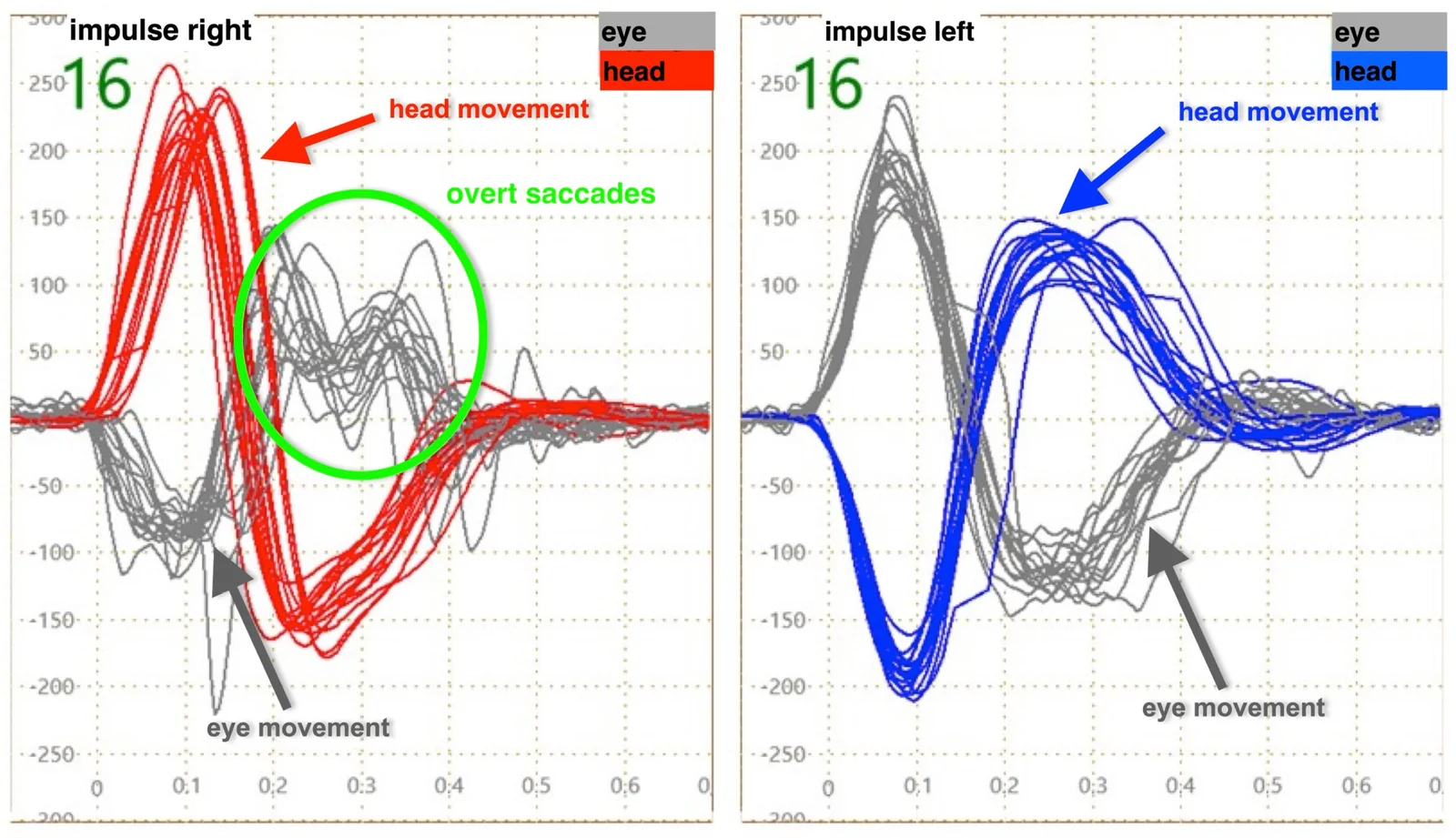
Illustrative presentation of the results of a pathological vHIT showing head impulses (number of impulses is given in green) to the right with reduced gain on the right and catch-up saccades on the right, while the left side shows normal results.

Mean (M) gain (±SD) was calculated for both sides at 40, 60, and 80 ms, with 2D/3D visualizations (time vs. velocity; gain vs. peak velocity; see Figure 1). Four clinicians conducted the tests; because of the retrospective design, it could not be reconstructed whether the same examiner performed the preoperative and the postoperative examination of a given patient. Catch-up saccades were visually assessed from 2D plots by a single experienced and blinded evaluator. The available recordings were randomly assigned for evaluation, and the evaluator was blinded to the pre- or postoperative status, surgical history, and side of implantation. Thus, the evaluator had no information as to whether a recording was obtained before or after CI surgery or which ear had been implanted. For the statistical analysis, catch-up saccades were classified as present or absent. A quantitative analysis of saccade characteristics, including amplitude, latency, or covert versus overt distribution, was not performed.

Catch-up saccades were additionally analyzed separately from vHIT gain, as they may represent a more robust parameter for detecting vestibular alterations, particularly in the presence of variable or potentially artefact-prone gain measurements [15,16]. The vHIT is classified as pathological if the gain is abnormal and refixation saccades are present. A vHIT gain of <0.8 was considered pathological, in accordance with established normative values and Bárány Society recommendations. Most studies specify a cutoff value for the gain of 0.79 or 0.8 [17,18,19]. For statistical analysis, the vHIT gain at 60 ms was selected a priori, because previous work suggested a lower susceptibility to certain goggle-slippage artefacts [16]. This does not imply that 60 ms is the uniquely most reliable instantaneous gain metric; test–retest work using the same device has reported the highest test–retest correlation for instantaneous gain at 80 ms [20].

The SVV test assesses otolith-related graviceptive processing by measuring deviations in a patient’s perception of vertical orientation. The static subjective visual vertical (SVV) was measured using a laser-projected line (Vertitest, INSTRUMENTATION S.A. DIFRA, Eupen, Belgium) in complete darkness without rotational stimulation to screen for otolith dysfunction. Participants adjusted the line to their perceived vertical across six trials, comprising three rotations from left to right and three from right to left, with randomized initial orientations to prevent anticipation of starting angle or direction. Deviations from true vertical were recorded in degrees together with an algebraic sign that encodes the alignment relative to the direction of rotation—a clockwise adjustment (left-to-right) that came to rest to the left of true vertical, and a counterclockwise adjustment (right-to-left) that came to rest to the right of true vertical—were both coded as negative, that is, a negative sign denotes an undershoot of the true vertical and a positive sign an overshoot. The sign was recorded to allow the direction of a pathological deviation to be described, but was not used in the analysis: The descriptive and inferential analyses were based on the absolute deviation from true vertical. For each participant and session, the three trials of each rotation direction were averaged, giving one value per direction; the statistical unit was therefore the participant and not the individual trial. The two rotation directions were analyzed separately and were not pooled, and no re-coding relative to the implanted side was applied; pathological findings are instead described as ipsilateral or contralateral to the implant in the Results section. A measurement was classified as pathological when the mean of the three trials of a given direction deviated by more than 2° from true vertical. The SVV was considered pathological if patients were unable to align the SVV within 2° of the true vertical. However, there is no generally accepted standard range for distinguishing between a normal SVV deviation and a vertical spatial perception disorder; the standard ranges in previous studies typically vary between ±2° [21,22,23], ±2.5° [24,25], and ±3° [26,27]. The postoperative SVV measurements were conducted during the patient’s postoperative hospital stay and therefore took place 3 to 5 days after surgery and before the patient’s discharge. The ±2° threshold was applied as the study definition of a pathological SVV. Because published normal ranges vary, the continuous SVV deviation was regarded as the outcome of primary interest and the categorical classification as a threshold-dependent secondary analysis.

### 2.2. Clinical Assessment of Dizziness and Nystagmus

In addition, dizziness was recorded based on the patients’ reports during routine postoperative clinical examinations. No standardized or validated questionnaire was used due to the retrospective design of the study. The presence of nystagmus was assessed using Frenzel goggles. Only clearly observable spontaneous nystagmus of clinical relevance was documented in the patient records. No predefined quantitative threshold (e.g., slow-phase velocity) was applied. Because dizziness was extracted from routine clinical documentation, the absence of a documented complaint does not represent a systematic negative assessment.

### 2.3. Radiological Assessment

The insertion depth of the cochlear implant electrode array was measured on postoperative CT scans by a single radiologist using multiplanar reconstruction and was defined as the linear intracochlear length from the round window to the most apical electrode contact. The radiological measurements were performed as part of the routine postoperative imaging evaluation and independently of the vestibular testing; formal blinding to the vestibular results was, however, not documented prospectively. Repeated measurements were not available, so intra- and inter-rater reliability could not be assessed. Angular insertion depth, which is anatomically more informative because cochlear dimensions vary considerably between individuals [28], could not be reconstructed retrospectively; linear insertion depth is therefore reported, and this is acknowledged as a limitation. Insertion depth could be determined in 41 of the 43 participants (23 right-sided and 18 left-sided implants); in two participants, the postoperative position check had been performed as cone-beam volume tomography, which could not be validated radiologically. All measurements were taken from the round window, which was the surgical entry point in every case, and were reviewed and validated by one experienced radiologist, so that a consistent reference point was used throughout.

All collected data were statistically analyzed to determine significant changes in vestibular function following CI surgery. For analysis, two groups were defined: (1) the overall study cohort including all patients, and (2) a subgroup excluding patients with a history of prior otologic surgery. The latter was analyzed as a sensitivity analysis to assess the potential influence of previous surgical interventions on vestibular function. The primary outcome measure was the vHIT gain at 60 ms obtained by vHIT, as well as the presence of compensatory catch-up saccades as a complementary parameter of semicircular canal function. For the present report, the analyses were structured hierarchically. The primary analysis was the paired preoperative-to-postoperative comparison of the horizontal vHIT gain at 60 ms in the implanted ear: within each participant, the gain of the ear that was subsequently implanted was compared with the gain of the same ear after surgery. This comparison was carried out separately for right- and left-sided recipients, because the distributional assumptions differed between the two subgroups and different tests were therefore required; the two side-specific results were not pooled into a single overall estimate. Key secondary analyses were the occurrence of catch-up saccades and the continuous SVV deviation. The comparison between the implanted and the non-implanted ears, all direction-specific saccade analyses, the categorical ±2° SVV classification, the correlations with electrode insertion depth, and the sensitivity analysis excluding patients with prior otologic surgery were exploratory and are reported as such.

### 2.4. Statistical Analysis

Secondary outcome measures included SVV deviation (in degrees), subjective dizziness reports, and electrode insertion depth. The normality of data distribution was assessed using the Shapiro–Wilk test. For normally distributed data, paired *t*-tests were used to compare pre- and postoperative values. In cases of non-normal distribution or small sample size (n < 30), the Wilcoxon signed-rank test was applied as a non-parametric alternative. The selection of statistical tests was based on data distribution characteristics and sample size considerations. For the primary comparison of the implanted ear, the Shapiro–Wilk test confirmed normality in the right-sided subgroup, for which a paired *t*-test was used, but rejected it in the left-sided subgroup, for which the Wilcoxon signed-rank test was used. Because the two subgroups were therefore analyzed with different tests, no pooled inferential estimate across implantation sides is reported. Mean paired changes are given with 95% confidence intervals and Cohen’s dz wherever a parametric test was applied; for the non-parametric comparisons, the test statistic and exact *p*-value are reported, as no corresponding interval estimate is available. For correlations, the coefficient, the number of observations and the 95% confidence interval obtained by Fisher’s z transformation are reported.

Analyses were performed for the complete study cohort and repeated as a sensitivity analysis in the subgroup of patients without previous otologic surgery. For vHIT, intra-individual preoperative-to-postoperative changes were additionally assessed separately according to the side of CI surgery. Pre- and postoperative vHIT gain values at 60 ms were compared separately for the right and left CI sides using paired *t*-tests for approximately normally distributed data and Wilcoxon signed-rank tests for non-normally distributed data. In addition, Pearson correlation analyses were performed to assess the association between pre- and postoperative vHIT gain values. These comparisons were performed separately within each implantation-side group. No formal test of a time × implantation-side interaction and no direct comparison of individual preoperative-to-postoperative change scores between the two implantation-side groups were performed. A statistically significant change within one side-specific subgroup, together with a non-significant change within the other, does not establish a statistically significant difference between the subgroups, and no such difference is claimed in this report.

The occurrence of catch-up saccades on the ipsilateral (implanted) side was classified according to pre- and postoperative status as absent/absent (unchanged), absent/present (new postoperative), present/absent (resolved), or present/present (persistent). Paired preoperative-to-postoperative changes in ipsilateral catch-up saccade status were assessed using an exact two-sided McNemar test, separately for right- and left-sided CI. The same preoperative-to-postoperative analysis was repeated in the subgroup of patients without previous ear surgery. In addition, postoperative differences in the occurrence of catch-up saccades according to the CI side were assessed using the Wald H_0_ test, with Agresti–Caffo and Newcombe confidence intervals calculated for the difference in proportions. The association between electrode insertion depth and the occurrence of catch-up saccades was assessed using the Mann–Whitney U test. No formal intra-rater or inter-rater reliability assessment was performed. The comparison of postoperative catch-up saccades according to the CI side and all direction-specific analyses were restricted to ears in which no catch-up saccade was present preoperatively in the respective direction; they therefore estimate the incidence of newly developed saccades rather than their overall prevalence. Ears with a catch-up saccade already present before surgery were excluded from these comparisons because a newly developed saccade cannot be defined for them. This restriction is the reason why the denominators of the postoperative and direction-specific comparisons (16 for left-sided CI and 15 for right-sided CI) are smaller than those of the paired preoperative-to-postoperative analyses (19 and 17, respectively).

Pearson correlation analyses were used to examine linear relationships between continuous variables, including vHIT gain, SVV deviation, and electrode insertion depth, assuming approximate normality. Pre- and postoperative SVV values were compared using paired *t*-tests where appropriate; non-parametric comparisons were performed using the Wilcoxon signed-rank test. SVV results were additionally analyzed separately according to the CI side and measurement direction. For the categorical analysis of pathological versus non-pathological SVV measurements, paired pre- and postoperative proportions were compared using the McNemar test, with Bonett–Price, Newcombe, and Wald confidence intervals calculated for the difference in proportions.

Pearson correlation analyses were used to assess the relationship between electrode insertion depth and vHIT gain, as well as between electrode insertion depth and SVV deviation. *p*-values less than 0.05 were considered significant. No formal correction for multiple testing was applied. A blanket Bonferroni correction was considered inappropriate for an analysis of this size and exploratory character, as it would substantially increase the type II error rate without resolving the underlying question of interpretation [29,30]. Multiplicity was instead addressed through the analysis hierarchy described above: Only the primary analysis is interpreted inferentially, whereas all exploratory results are reported descriptively and are explicitly labelled as hypothesis-generating. Post hoc “observed power” calculations were deliberately omitted, as they are a direct function of the observed *p*-value and provide no additional information [31].

Statistical analyses and graphical representations were performed using IBM SPSS Statistics, version 29.0.2.0 (IBM Corp., Armonk, NY, USA).

## 3. Results

A total of 43 patients were included in the study. The mean age of participants was 59 ± 15 years (range: 22–90 years), with a nearly equal distribution of 20 male (46.5%) and 23 female (53.5%) patients. All patients fulfilled the institutional criteria for CI corresponding to severe-to-profound sensorineural hearing loss with insufficient benefit from conventional hearing rehabilitation.

Most patients presented with bilateral (n = 20) or asymmetric (n = 20) severe-to-profound hearing loss. A small number of patients (n = 3) exhibited unilateral deafness with normal hearing on the contralateral side.

Primary analyses were performed comparing pre- and postoperative vestibular function, with additional comparisons between implanted and non-implanted ears.

**Figure 2:**
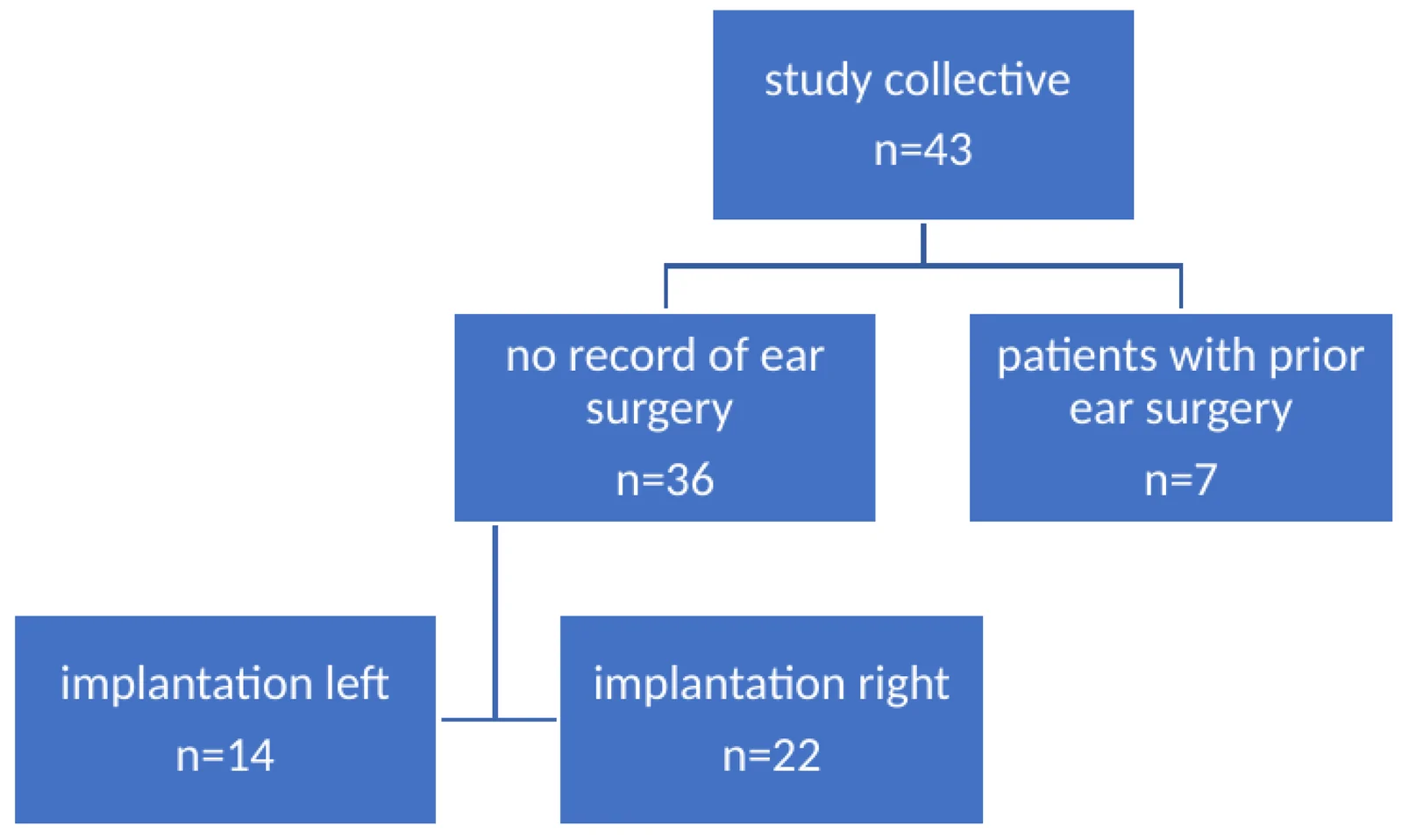
Flowchart of the study groups.

Nineteen patients underwent left-sided and 24 patients right-sided CI. Seven patients had undergone ear surgery before, five of them had CI on the opposite side. One patient had a surgery due to acoustic neuroma on the implantation side, and one had previously undergone a tympanoplasty. All relevant analyses were additionally performed for the subgroup of patients without prior ear surgery (n = 36) as a sensitivity analysis (see Figure 2). In 27 cases, the electrodes from Advanced Bionics (Valencia, CA, United States) were implanted; in two cases, the electrodes from Med-El were used, and in 14 cases, Cochlear electrodes were used. The electrode arrays used were as follows: for Advanced Bionics, ten HiRes Ultra, seven HiRes 90K, and ten HiRes Ultra with a HiFocus thin and flexible array; for Cochlear (Macquarie University, NSW, Australia), three CI422 and eleven CI522 arrays, both of the slim straight lateral-wall type; and for MED-EL (Innsbruck, Austria), one Concerto Mi1200 with a Flex array and one Flex28 array. As array geometry, length, and stiffness differ substantially within and between manufacturers [14], the insertion-depth analyses are confounded by heterogeneous electrode designs and are reported as exploratory.

According to patient records, no one reported symptoms of dizziness prior to surgery. As for the postoperative symptom data, this information derives from routine clinical documentation rather than from a systematic enquiry, so the absence of a documented complaint cannot be equated with the absence of symptoms.

Insertion depth was measurable in 41 of the 43 participants and averaged 20 mm (range: 16–25 mm); the shortest insertion of 16 mm occurred once, the most frequent value was 18 mm (12 participants) and the maximum of 25 mm was documented three times. Postoperative dizziness was documented in the records of five patients (11.6%); in two cases (4.7%), nystagmus was clinically observed during the postoperative rounds (using Frenzel goggles to suppress fixation). One of them showed an irritative nystagmus, whereas the other one exhibited paretic nystagmus. Electrode insertion depth was not significantly associated with the occurrence of newly developed ipsilateral catch-up saccades (Mann–Whitney U test: total cohort n = 30, *p* = 0.423; subgroup n = 25, *p* = 0.894), with postoperative dizziness (n = 41, *p* = 0.403), with spontaneous nystagmus (n = 41, *p* = 0.351) or with any of the postoperative SVV measures (Pearson correlation, n = 41, all *p* > 0.05). The correlations with postoperative vHIT gain are reported in Section 3.3.

### 3.1. Video Head Impulse Test (vHIT)

**Figure 3:**
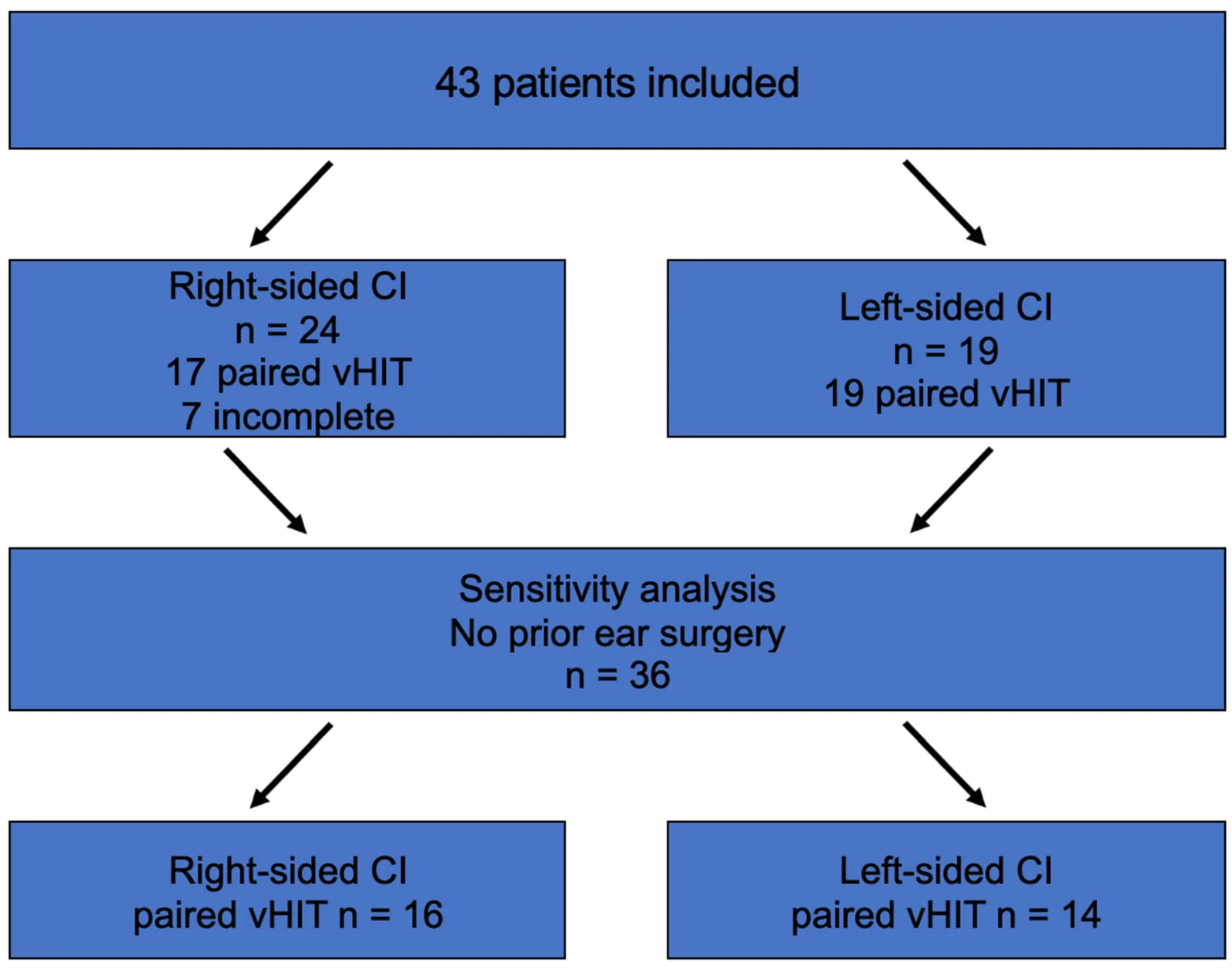
Flowchart of patient inclusion for the vHIT analysis. Of the 43 patients included in the study, seven were excluded from the vHIT analysis because paired pre- and postoperative vHIT measurements were unavailable (three patients lacked both examinations, three lacked postoperative measurements only, and one lacked the preoperative examination). Consequently, 36 patients with paired vHIT data were available for analysis. These comprised 17 patients with right-sided CI and 19 patients with left-sided CI. Subgroup analysis excluding patients with previous otologic surgery included 30 patients with paired vHIT measurements (16 right-sided and 14 left-sided CI).

Seven patients were excluded from the vHIT analysis because paired pre- and postoperative examinations were unavailable. Specifically, three patients had neither pre- nor postoperative vHIT measurements, three had only preoperative measurements, and one had only postoperative measurements. Thus, paired vHIT data were available for 36 of the 43 included patients. The exclusion was based solely on data availability and was independent of the CI side. Of the 24 patients who underwent right-sided CI, paired vHIT data were available for 17 patients. Of the 19 patients with left-sided CI, paired data were available for all 19 patients. In the sensitivity analysis excluding patients with previous otologic surgery, paired vHIT data were available for 16 right-sided and 14 left-sided CI recipients (see Figure 3). All seven patients without paired vHIT data had undergone right-sided implantation; missingness was therefore asymmetric with respect to the CI side. A missing-at-random assumption cannot be established for these data, and the side-stratified comparisons are correspondingly vulnerable to selection bias.

Primary analysis. Complete paired pre- and postoperative vHIT measurements of the implanted ear were available for 36 of the 43 participants (17 right-sided and 19 left-sided CI). The prespecified primary comparison of the implanted ear was performed separately for the two implantation sides, because the Shapiro–Wilk test confirmed normality of the paired differences in the right-sided subgroup but rejected it in the left-sided subgroup, so that a paired *t*-test and a Wilcoxon signed-rank test were required, respectively. After right-sided CI, the gain at 60 ms was 1.52 ± 0.37 preoperatively and 1.59 ± 0.63 postoperatively; the mean paired change was +0.07 (95% CI: −0.41 to 0.56; paired *t*-test t(16) = 0.32, two-sided *p* = 0.754; Cohen’s dz = 0.08). After left-sided CI, the median gain fell from 1.41 (IQR: 0.51) to 1.07 (IQR: 0.41), corresponding to means of 1.44 and 1.04, respectively (Wilcoxon signed-rank test z = −3.02, two-sided *p* = 0.003). Because the two subgroups were analyzed with different tests, the two results were not pooled into a single overall estimate, and no pooled confidence interval or effect size is available. For the right-sided comparison, the confidence interval is compatible with a reduction in gain of up to approximately 0.41 units and with an increase of up to approximately 0.56 units, so a clinically relevant deterioration cannot be excluded on the basis of these data; for the left-sided comparison, no interval estimate is available, and the size of the change can therefore not be quantified with a measure of precision. All analyses reported below are secondary or exploratory.

**Figure 4:**
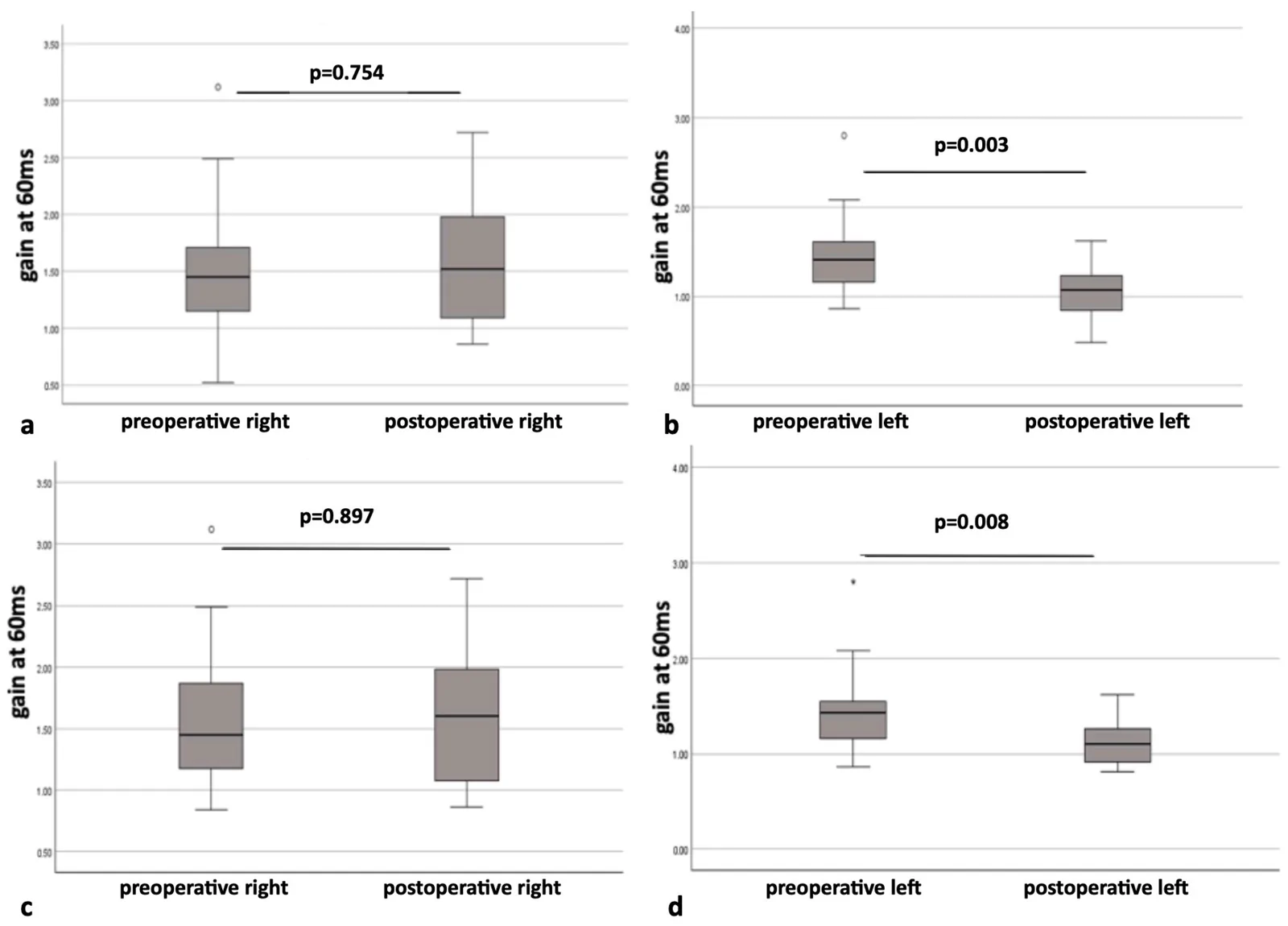
Depiction of pre- and postoperative VOR testing gain results at 60 ms of the right side (n = 17, *p* = 0.754) (**a**) and the left side (n = 19, *p* = 0.003) (**b**), as well as the results of VOR testing without prior ear surgery, right (n = 16, *p* = 0.897) (**c**) and left side (n = 14, *p* = 0.008) (**d**). *p*-values are given above the box plots. Data are presented as box-and-whisker plots, outliners in the data are marked as small circles or stars. The horizontal line within each box represents the median, and the box represents the interquartile range (IQR). The whiskers extend to the most extreme values within 1.5 × IQR from the first and third quartiles; individual points beyond the whiskers represent outliers. *p*-values were calculated using the Wilcoxon signed-rank test (**b**,**d**) or *t*-test (**a**,**c**) and are shown above the respective plots.

The side-specific primary comparisons are reported above. Preoperative and postoperative gain values were not significantly correlated on the right side (Pearson r = −0.22; 95% CI: −0.63 to 0.29; n = 17; *p* = 0.400). Similar results were found for the subgroup analysis of patients without surgery before CI with z = 0.13, *p* = 0.897 for right-sided CI (n = 16; preoperative mean: 1.58, median: 1.45, IQR: 0.78; postoperative mean: 1.61, median: 1.60, IQR: 0.91) and z = −2.67, *p* = 0.008 for left-sided CI (n = 14; preoperative mean: 1.48, median: 1.43, IQR: 0.42; postoperative mean: 1.22, median: 1.10, IQR: 0.39). Because these are separate within-group analyses that were not accompanied by a formal comparison between implantation sides, and because all gain values are affected by the systematic measurement offset discussed below, they are reported as exploratory and are not interpreted as evidence of a side-specific biological effect. The results are shown in Figure 4.

Further, the within-participant comparison of the implanted and the non-implanted ear showed no significant differences in either cohort. Notably, vHIT gain values were generally above 1.0 both pre- and postoperatively and showed considerable variability. Values of this magnitude are not physiologically plausible and most probably reflect a systematic measurement offset rather than vestibular hyperfunction. Because the preoperative-to-postoperative comparisons were performed separately within each implantation-side group, and neither an interaction test nor a between-group comparison of change scores was carried out, the divergent significance pattern between the two subgroups does not demonstrate a statistically significant difference between implantation sides.

**Figure 5:**
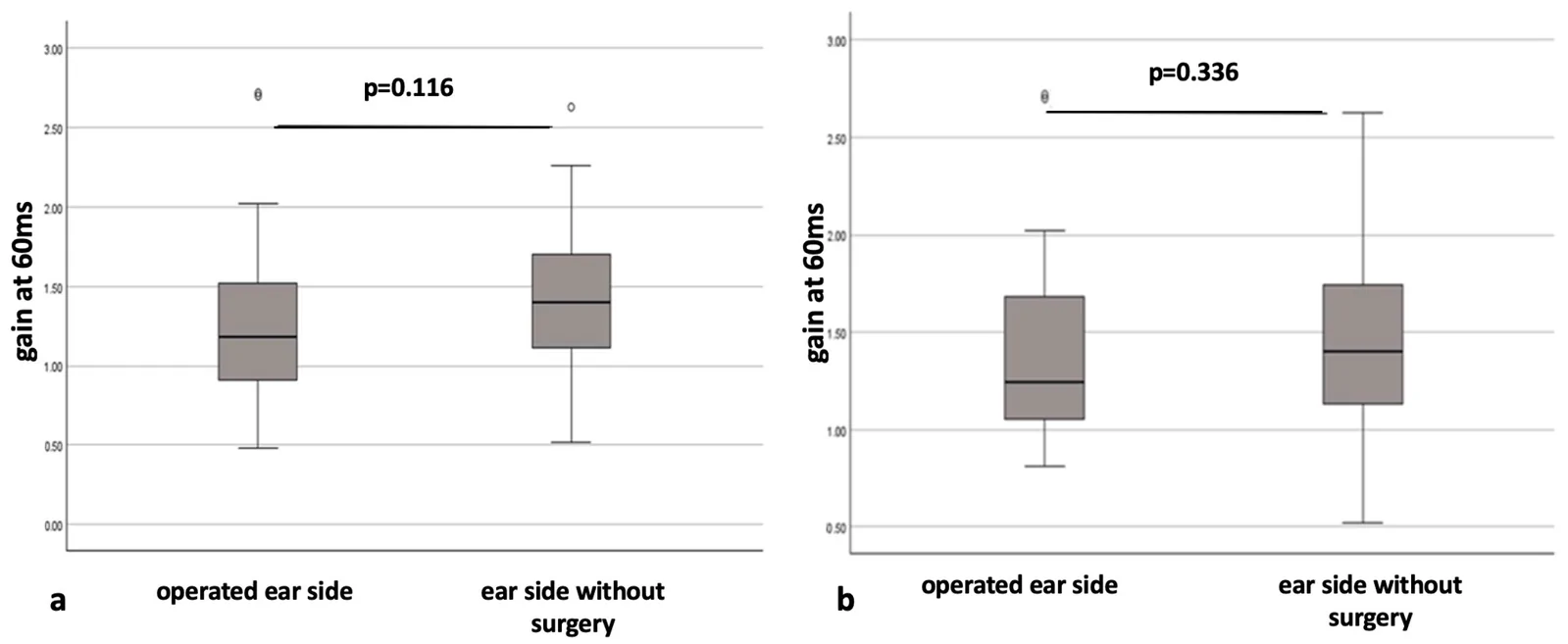
Comparison of postoperative vHIT gain values at 60 ms between the operated and non-operated ear sides. (**a**) Complete study group (n = 37, *p* = 0.116). (**b**) Subgroup of patients without previous ear surgery (n = 30, *p* = 0.336). Data are presented as box-and-whisker plots. The horizontal line within each box represents the median, and the box represents the interquartile range (IQR). The whiskers extend to the most extreme values within 1.5 × IQR from the first and third quartiles; individual points beyond the whiskers represent outliers. *p*-values were calculated using the paired *t*-test and are shown above the respective plots.

Implanted versus non-implanted ear. This comparison concerned the postoperative gain values of the two ears of the same participant, rather than the preoperative-to-postoperative change; it was performed as a paired analysis within participants and included the 37 participants for whom postoperative vHIT data of both ears were available. The mean postoperative gain at 60 ms was 1.29 in the implanted ear and 1.43 in the non-implanted ear (paired *t*-test, *p* = 0.116; Cohen’s d = −0.20); the gain values of the two ears were only weakly correlated (Pearson r = 0.23, *p* = 0.084). In the subgroup without previous ear surgery (n = 30), the corresponding values were 1.38 and 1.43 (*p* = 0.336; Cohen’s d = −0.08). Three participants had single-sided deafness with normal hearing contralaterally and 20 had asymmetric hearing loss, so the non-implanted ear cannot be regarded as an unaffected control: pre-existing interaural vestibular asymmetry and central compensation may affect both ears, and no adjustment for pre-existing asymmetry was made. This comparison is therefore exploratory and is not evidence of an absence of a postoperative effect. The results are shown in Figure 5.

Among patients with paired pre- and postoperative vHIT assessments, 6 of 17 patients (35.3%) with right-sided CI and 8 of 19 patients (42.1%) with left-sided CI developed new postoperative ipsilateral catch-up saccades that were absent preoperatively. Ipsilateral catch-up saccades were persistent in 2 patients (11.8%) after right-sided CI and 2 patients (10.5%) after left-sided CI. No right-sided saccades resolved postoperatively, whereas 1 patient (5.3%) with left-sided CI showed resolution of a preoperatively present ipsilateral saccade. The remaining patients had no ipsilateral catch-up saccades at either assessment (right-sided CI: 9/17, 52.9%; left-sided CI: 8/19, 42.1%). The complete preoperative-to-postoperative classification is presented in Table 1. Across both implantation sides, 14 of the 36 participants with paired data (38.9%) developed new ipsilateral catch-up saccades that had been absent preoperatively. This is a substantial proportion and is one of the reasons why the non-significant gain analyses must not be read as demonstrating preserved horizontal vestibulo-ocular function.

An exact two-sided McNemar test demonstrated a significant preoperative-to-postoperative change in ipsilateral catch-up saccade status in the total cohort for both right-sided CI (*p* = 0.031) and left-sided CI (*p* = 0.039).

In the sensitivity analysis excluding patients with previous ear surgery, 5 of 16 patients (31.3%) with right-sided CI and 7 of 14 patients (50.0%) with left-sided CI developed new postoperative ipsilateral catch-up saccades. Ipsilateral saccades persisted in 2 patients (12.5%) after right-sided CI and 1 patient (7.1%) after left-sided CI, while 1 patient (7.1%) with left-sided CI showed resolution of a preoperatively present saccade. The exact two-sided McNemar test did not demonstrate a statistically significant preoperative-to-postoperative change in the subgroup (right-sided CI: *p* = 0.063; left-sided CI: *p* = 0.070).

A separate exploratory analysis compared postoperative catch-up saccades according to their direction and CI side. As described in the Methods section, these comparisons were restricted to ears without a preoperative catch-up saccade in the respective direction, so that they estimate the incidence of newly developed saccades; the five participants with a preoperatively present saccade (three with left-sided and two with right-sided CI) were therefore not eligible for this analysis, which reduces the denominators from 19 and 17 to 16 and 15. Newly developed postoperative catch-up saccades were observed in 8 of 16 patients (50.0%) after left-sided CI and in 6 of 15 patients (40.0%) after right-sided CI. The overall difference between CI sides was not statistically significant (Wald H_0_ test: z = 0.559, *p* = 0.576); the corresponding Agresti–Caffo and Newcombe confidence intervals included zero.

A direction-specific analysis revealed different patterns for right- and left-directed catch-up saccades. Right-directed catch-up saccades occurred in 2 of 16 patients (12.5%) after left-sided CI and in 6 of 15 patients (40.0%) after right-sided CI. This difference did not reach statistical significance in the two-sided analysis (Wald H_0_ test: z = 1.79, *p* = 0.080; Agresti–Caffo 95% CI: −0.045 to 0.536; Newcombe 95% CI: −0.035 to 0.534).

In contrast, left-directed catch-up saccades occurred in 8 of 16 patients (50.0%) after left-sided CI and in 1 of 15 patients (6.7%) after right-sided CI. This difference was statistically significant (Wald H_0_ test: z = −2.656, two-sided *p* = 0.008; Agresti–Caffo 95% CI: −0.660 to −0.105; Newcombe 95% CI: −0.660 to −0.114). Thus, the significant finding was restricted to left-directed catch-up saccades and did not reflect a significant difference in the overall occurrence of catch-up saccades between left- and right-sided CI. Given the small sample size and the qualitative assessment of catch-up saccades, this direction-specific finding should be interpreted as exploratory.

The two examinations were performed at systematically different times after surgery. Postoperative SVV was obtained a median of 3 days after surgery (IQR: 2 to 4 days; range: 1 to 44 days); 4 participants (9.3%) were examined on day 1, 11 participants (25.6%) were examined on day 2, 14 participants (32.6%) were examined on day 3, 11 participants (25.6%) were examined on day 4, one patient each (2.3%) was examined on days 5, 43, and 44, and the last two participants were examined at outpatient follow-up. Postoperative vHIT data were available for 37 of the 43 patients and were obtained much later and far more heterogeneously: 9 participants (24.3%) were examined within the first three days during the initial inpatient stay, 2 participants (5.4%) were examined between days 30 and 40, 20 participants (54.1%) were examined between days 40 and 50, and 6 participants (16.2%) were examined after day 50, the longest intervals being 92 and 140 days; the median interval fell within the 40-to-50 day range. Timing was not analyzed separately according to implantation side, and the association between the postoperative interval and the preoperative-to-postoperative change in vHIT gain was not examined. Because postoperative SVV and vHIT were thus obtained at systematically different intervals after surgery, the two measures do not describe vestibular function at the same biological time point. An exploratory analysis of the association between the postoperative interval and the change in vHIT gain was not performed.

The reasons for this variability could not be determined but may relate to the more demanding nature of vHIT testing in the early postoperative period.

### 3.2. Subjective Visual Vertical (SVV)

**Figure 6:**
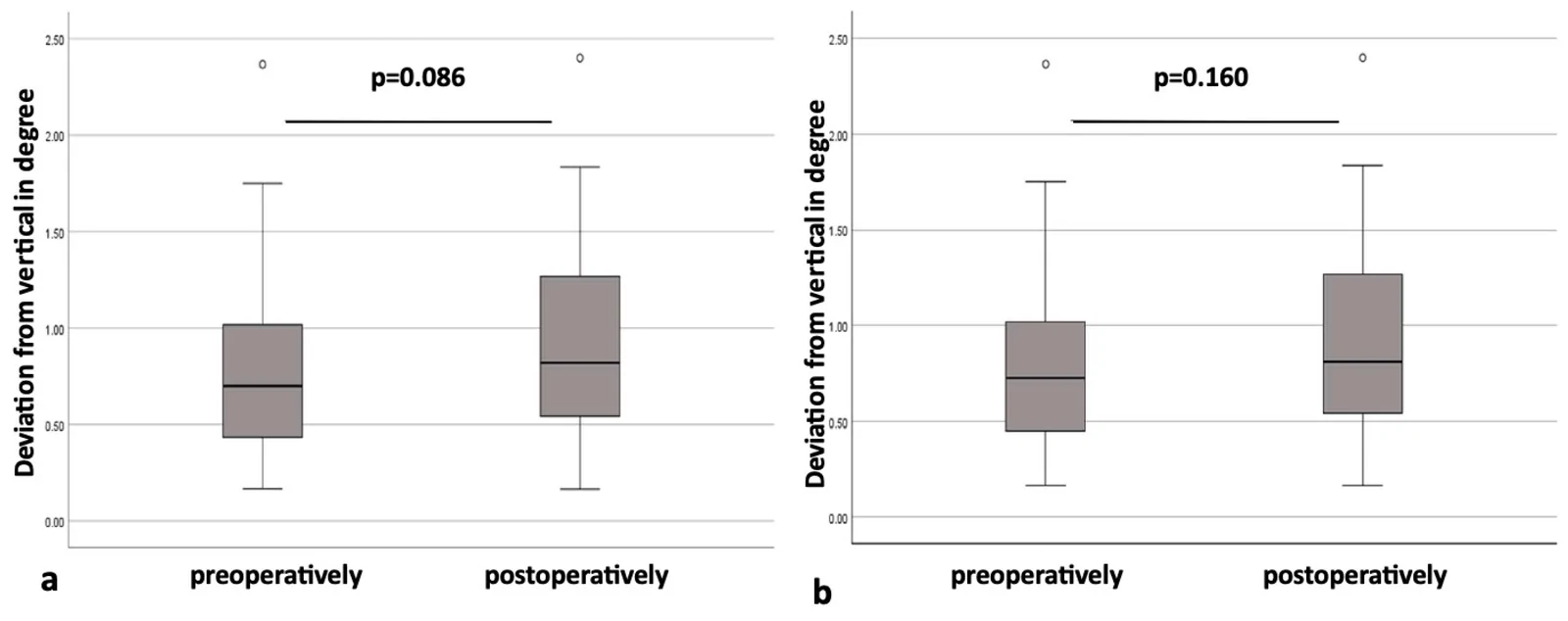
Comparison of preoperative and postoperative SVV values in degrees between the operated and non-operated ear sides. (**a**) Complete study group (n = 43, *p* = 0.086). (**b**) Subgroup of patients without previous ear surgery (n = 36, *p* = 0.160). Data are presented as box-and-whisker plots. The horizontal line within each box represents the median, and the box represents the interquartile range (IQR). The whiskers extend to the most extreme values within 1.5 × IQR from the first and third quartiles; individual points beyond the whiskers represent outliers. *p*-values were calculated using the paired *t*-test and are shown above the respective plots.

Postoperative SVV tests were mostly conducted within the first five days after CI surgery, with the majority occurring on day 3. Pathological SVV values (i.e., inability to align within 2° of true vertical) were documented in a minority of participants in both groups. The continuous outcome was the mean absolute deviation from true vertical, averaged over the three trials of each rotation direction. It was 0.77 ± 0.47° preoperatively and 0.92 ± 0.51° postoperatively (n = 43; paired *t*-test *p* = 0.086; Cohen’s d = −0.21); pre- and postoperative values were essentially uncorrelated (Pearson r = 0.03, *p* = 0.436). In the subgroup without previous ear surgery, the corresponding values were 0.80 ± 0.47° and 0.92 ± 0.51° (n = 36; *p* = 0.160; Cohen’s d = −0.17). Normality was assessed with the Shapiro–Wilk test; because it was not confirmed but the sample exceeded 30 and no outliers were present, a paired *t*-test was applied in accordance with the central limit theorem. The paired categorical comparison of pathological versus non-pathological classification was performed with the McNemar test. Neither analysis detected a statistically significant postoperative change in otolith-related graviceptive processing as assessed by SVV, although the direction of the numerical change was towards larger deviations. Although postoperative values were numerically slightly higher, the difference was small and did not meet the prespecified significance threshold of *p* < 0.05. Preoperatively, three participants had pathological SVV values, all of whom were normal postoperatively. Postoperatively, seven patients (16.3%) exhibited pathological SVV values, in five of them (11.6%) on the implanted side, with a higher proportion among those with left-sided CI. After right-sided CI, one person (4.2%) showed pathological SVV measurement postoperatively, whereas six pathological measurements (31.6%) were found after left-sided CI. In four of these cases, the measurement was pathological in the ipsilateral side, in one case on the contralateral side, and one patient showed pathological results for both sides. The results are depicted in Figure 6. These seven participants exceeded the prespecified ±2° study threshold; the observed values may nevertheless fall within the broader normative limits of ±2.5° to ±3° applied in some previous studies. The categorical classification is therefore strongly threshold-dependent; a sensitivity analysis using ±2.5° or ±3° was not performed, and the continuous SVV analysis should accordingly be regarded as the primary SVV result.

A directional analysis (right-to-left vs. left-to-right) revealed no consistent pattern of pathological changes. Among the 19 participants with left-sided CI, pathological values in the right-to-left direction increased from 1 preoperatively to 5 postoperatively (exact McNemar test z = −1.63, two-sided *p* = 0.102) and in the left-to-right direction from 0 to 2 (z = −1.41, two-sided *p* = 0.157). Neither difference met the prespecified significance threshold and both are reported descriptively.

**Figure 7:**
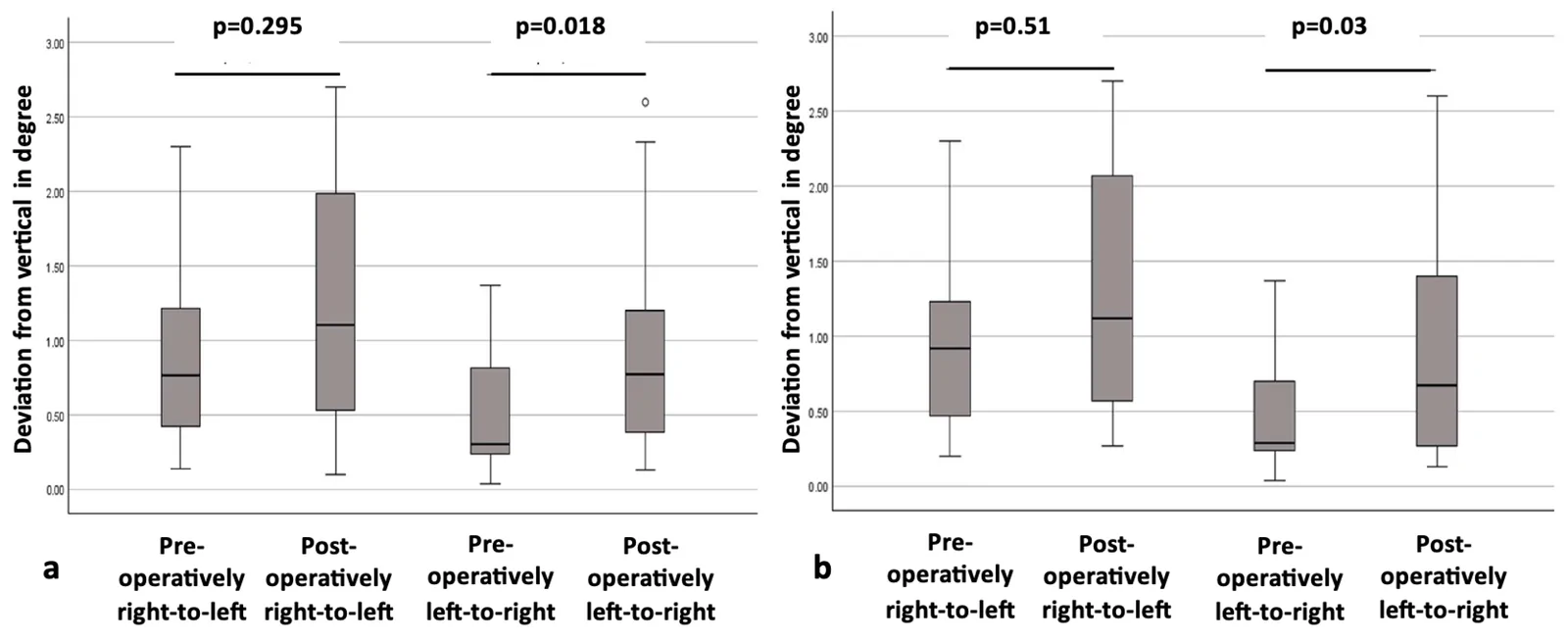
Comparison of preoperative and postoperative SVV values in degrees in directional analysis in patients with CI on the left side. (**a**) Complete study group (n = 43) SVV measurements taken from right to left showed no significant difference between preoperative and postoperative values (*p* = 0.295). For the direction from left to right, SVV measurements showed significant difference between preoperative and postoperative values (*p* = 0.018). (**b**) Subgroup of patients without previous ear surgery (n = 36). SVV measurements taken from right to left showed no significant difference between preoperative and postoperative values (*p* = 0.51). For the direction from left to right, SVV measurements showed significant difference between preoperative and postoperative values (*p* = 0.03). *p*-values are given above the box plots. Data are presented as box-and-whisker plots with individual paired measurements connected by lines. The horizontal line within each box represents the median, and the box represents the interquartile range (IQR). The whiskers extend to the most extreme values within 1.5 × IQR from the first and third quartiles; individual points beyond the whiskers represent outliers. *p*-values were calculated using the Wilcoxon signed-rank test and are shown above the respective plots.

In patients without prior ear surgery, the findings were similar: six (16.7%) showed postoperative pathological SVV values, predominantly on the ipsilateral side. Again, no significant differences were found between pre- and postoperative measurements (see Figure 7 for details).

### 3.3. Cochlear Implant Insertion Depth and vHIT

The mean insertion depth measured on postoperative CT scans was 20 mm (range: 16–25 mm). After right-sided CI, the postoperative vHIT gain at 60 ms showed a negative Pearson correlation with insertion depth (r = −0.32, 95% CI: −0.59 to 0.01, n = 36, *p* = 0.056); the confidence interval is wide and includes zero, so this is an imprecise exploratory estimate rather than a demonstrated association. After left-sided CI, the corresponding correlation was very weak (r = −0.09, 95% CI: −0.40 to 0.25, n = 36, *p* = 0.620). In the sensitivity analysis excluding participants with previous ear surgery, the right-sided correlation reached nominal significance (r = −0.37, 95% CI: −0.64 to −0.01, n = 30, *p* = 0.046), whereas the left-sided correlation remained negligible (r = −0.04, *p* = 0.825). This isolated nominally significant result arises from one of many exploratory correlations, was not corrected for multiple testing and was no longer apparent when the reported measurement uncertainty of the gain values was included (r = −0.17, *p* = 0.378 and r = −0.21, *p* = 0.276); it should therefore not be regarded as evidence of an effect of insertion depth. Because insertion depth is reported in millimeters rather than as angular insertion depth, because electrode array design was heterogeneous and unevenly distributed between manufacturers, because the measurement was made by a single radiologist without a reliability assessment, and because the sample sizes were small and the confidence intervals were wide, no statistically significant association was detected in this exploratory and methodologically limited analysis. This must not be interpreted as evidence that insertion depth is unrelated to vestibular outcomes.

## 4. Discussion

The findings of this study are in line with previous reports suggesting that CI may be associated with subtle alterations in vestibular test results, predominantly involving the horizontal semicircular canal and graviceptive pathways [8,10]. While histopathological studies indicate that the saccule is most frequently affected due to its anatomical proximity to the cochlea [7], functional assessments such as vHIT and SVV primarily reflect horizontal semicircular canal function and graviceptive pathway integrity, respectively, and do not assess saccular function.

Our data partially align with previous findings regarding postoperative vestibular alterations, particularly with respect to changes in vHIT gain. However, in contrast to some prior studies [8,10], we did not observe statistically significant changes in SVV values, suggesting that no significant postoperative alteration in otolith-related graviceptive processing was detected by SVV.

The comparison of pre- and postoperative vHIT data revealed no significant change in patients with right-sided CI, whereas a reduction in postoperative vHIT gain was observed in the left-sided subgroup, although gain values remained above the pathological threshold. As set out in the Methods section, these are separate within-group analyses that were not accompanied by a formal comparison between implantation sides. One limitation in evaluating our results lies in the overall elevated pre- and postoperative vHIT gain values, with mean values exceeding 1, as well as the high standard deviation reported by the device. The causes and potential influencing factors for this are diverse. A key factor could be a loosely fitting video goggle, which may lead to the phenomenon known as “goggle slippage”. Suh et al. demonstrated that the backward slippage of the video goggles during head movement can lead to an increased vHIT gain and may result in an overcompensation of eye movements. Furthermore, insufficient strap tension of the video goggles can impair the precision of the correlation between ocular and cranial movements, which can also lead to an increased gain. These changes were particularly observed at 40 ms and 80 ms in the study by Suh et al., whereas the gain at 60 ms remained relatively stable under various conditions [16]. Consequently, the observed postoperative reduction in left-sided vHIT gain cannot be interpreted unequivocally as a true vestibular deficit, as differences in goggle fit, calibration, or other session-specific measurement conditions between pre- and postoperative examinations may also have contributed to the observed change. Therefore, the primary outcome should be interpreted as an exploratory signal compatible with, but not specific to a biological postoperative alteration in horizontal semicircular canal function. Contemporary normative data acquired with the same device indicate that horizontal VOR gain in healthy adults clusters closely around 1.0 [32], and video-oculography studies have shown that markedly elevated gain values are more consistent with calibration or measurement problems than with physiological hyperfunction, particularly when they occur without corresponding refixation saccades [33]. The preoperative values of approximately 1.4 to 1.6 observed here therefore most probably reflect a systematic measurement offset. Consequently, the observation that postoperative gain values remained above the conventional pathological threshold of 0.8 offers only limited reassurance, since a systematic upward offset would also shift values away from that threshold; the absolute gain values reported here should accordingly not be read as physiological estimates. It should also be noted that the pathological threshold of 0.8 was established mainly for regression-based or area-under-the-curve gain and has not been validated specifically for the EyeSeeCam instantaneous gain at 60 ms used here. In combination with the systematic upward offset, this means that the threshold cannot be meaningfully applied to the present measurements, and all statements previously based on gain values remaining above 0.8 have been removed from the manuscript. The preoperative-to-postoperative difference is accordingly best described as a change in the recorded vHIT metric, which is not necessarily equivalent to a biological reduction in horizontal semicircular canal function. The original recordings could in principle be re-reviewed for calibration error, goggle slippage and implausible impulses, and a sensitivity analysis after applying an explicitly justified quality-control criterion would be the appropriate way to address this; such a re-analysis was beyond the scope of the present revision and is stated here as a limitation.

Nevertheless, several observations suggest that the findings are unlikely to be solely explained by measurement artefacts. vHIT gain was analysed at 60 ms, the metric selected a priori for its reported lower susceptibility to goggle-slippage effects—although instantaneous gain at 80 ms has shown higher test–retest reliability with this device [20]—postoperative vHIT gain values remained consistently above the pathological threshold, and a higher frequency of left-directed catch-up saccades was observed in the left-sided subgroup. Catch-up saccades may provide complementary information on vestibulo-ocular function. In the present cohort, however, the overall occurrence of catch-up saccades did not differ significantly according to the CI side. The significant difference observed for left-directed saccades was restricted to a single saccade direction and should therefore be regarded as an exploratory finding rather than as independent evidence of generalized postoperative vestibular hypofunction. Because gain and saccade classification are derived from the same potentially artefact-prone examination, the saccade findings are not an independent corroboration of the gain change.

The within-subject comparison of CI versus non-CI ears also showed numerically lower postoperative vHIT values after CI, although this difference did not meet the prespecified significance threshold. While catch-up saccades were predominantly observed on the implanted side, occasional findings in the contralateral ear were also noted. These contralateral occurrences, although less frequent and not statistically significant, may be attributable to central vestibular compensation mechanisms or measurement variability.

The reduction in vHIT gain observed in the left-sided subgroup warrants cautious interpretation. It must be emphasized that this observation rests on separate within-group comparisons: a statistically significant preoperative-to-postoperative change in one subgroup together with a non-significant change in the other does not demonstrate a statistically significant difference between implantation sides. No time × implantation-side interaction was tested and no comparison of individual change scores between the groups was performed, so the present data do not establish a side-specific effect of CI. The available literature does not support a consistent left-right asymmetry of vestibular outcomes following CI. Rather, previous studies have predominantly reported postoperative vestibular changes on the implanted side compared with on the non-implanted side, supporting a possible ipsilateral effect of the surgical intervention [10,34,35]. Potential explanations for the left-sided finding in our cohort include pre-existing interaural vestibular asymmetry, individual anatomical or surgical factors, electrode-related characteristics, or random variation related to the limited sample size. In our cohort, no significant association was found between electrode insertion depth and vestibular measures. This finding is consistent with previous studies that likewise found no significant relationship between insertion depth and postoperative vestibular dysfunction, although differences in electrode design and imaging methodology limit direct comparability [36,37]. Furthermore, variability in vHIT measurements between examiners and testing conditions may contribute to apparent differences between sides [15]. Therefore, the observed reduction in left-sided CI should be regarded as an exploratory finding rather than evidence of a specific left-sided susceptibility and requires confirmation in larger prospective cohorts. In addition, all seven patients without paired vHIT data had undergone right-sided implantation, so the right-sided subgroup was both smaller and potentially selected; differential missingness alone could contribute to the divergent significance pattern between the two subgroups. Four clinicians performed the vHIT examinations, and appreciable between-examiner variability in gain has been described for this device, whereas the identification of catch-up saccades appears to be more reproducible. An examiner- or session-related origin of the observed left–right asymmetry can therefore not be excluded.

It is plausible that the saccule and utricle, given their anatomical proximity to the cochlea, may be affected by CI [7]. Studies have reported postoperative cVEMP alterations in 38–63% of cases, supporting this hypothesis [6,10,38]. However, our study did not find a significant association between CI and pathological SVV deviations. Although seven participants exceeded the prespecified ±2° study threshold, the observed deviations remained within the broader normative limits of up to ±3° reported in some previous studies [22,39,40]. A deviation towards the implanted ear has been interpreted as compatible with an altered utricular/graviceptive pathway asymmetry with reduced afferent input from the implanted side, whereas a deviation away from the implanted ear has been interpreted as compatible with the opposite pattern, possibly related to perilymphatic changes or postoperative endolymphatic hydrops [41,42]. Because the SVV reflects not only peripheral utricular receptor function but also graviceptive pathway conduction and central perceptual integration, and because oVEMP corroboration was not available, the direction of SVV deviation alone does not permit a receptor-specific diagnosis of utricular hypo- or hyperfunction, particularly in the early postoperative period. In our study, although some patients showed directional deviations toward the implanted side, these remained within the normal range and therefore do not indicate a clinically relevant alteration in otolith-related graviceptive perception. Truong et al. demonstrated in their study that the SVV deviated significantly by an average of 2.17° away from the implanted ear one day after CI surgery. The authors interpreted this as a pattern compatible with utricular hyperfunction on the implanted side. However, after six weeks, follow-up measurements had normalized and no longer showed significant differences compared to the preoperative values [42]. Secondary endolymphatic hydrops has also been proposed as a potential mechanism underlying vestibular alterations after CI. Perilymphatic leakage, inflammatory reactions, and a subsequent disturbance of inner-ear fluid homeostasis have been discussed as contributing factors [43,44]. Such alterations have been linked to postoperative vestibular changes [43,45]. As hydrops was not assessed in the present study, this mechanism remains hypothetical and is not given further interpretive weight here. Since conditions may normalize over time, another limitation of our study is that the postoperative examination period mainly fell between the first and fifth postoperative days, although two patients did not undergo an SVV examination until the 43rd and 44th days, respectively. The postoperative vHIT assessments were also performed at variable time points, which may have influenced the measured vestibular parameters and limited direct comparability between patients. Furthermore, no follow-up was conducted. Therefore, it remains unclear whether the observed postoperative changes, including the occurrence of catch-up saccades, represent transient vestibular alterations that may resolve through central compensation or persistent vestibular impairment. The absence of longitudinal follow-up limited the interpretation of their clinical relevance.

No apparent differences in vestibular outcomes were observed between different CI manufacturers in this cohort. However, due to the small and uneven group sizes (e.g., only two patients in the MED-EL group), no meaningful statistical comparison can be made, and these observations should be interpreted with caution.

Postoperative subjective dizziness and nystagmus were documented infrequently in this cohort after CI via the round window; because neither was assessed systematically, this cannot be interpreted as evidence that such symptoms are rare. In our cohort, postoperative dizziness was documented in the records of 11.6% of patients and nystagmus was clinically observed in 4.7%. Because these data were extracted from routine clinical documentation rather than obtained with a validated instrument, the absence of a documented symptom is not equivalent to a systematic negative assessment; these figures should therefore not be read as incidence estimates and may underestimate the true frequency of postoperative dizziness. These findings are in line with previous studies reporting a wide range of 2% to 47% for subjective dizziness, likely reflecting differences in assessment methods and study design [38,46]. The round window approach is considered safe and minimizes the risk of vestibular function loss caused by possible damage to the basilar membrane and endolymph leakage, resulting in fewer subjective vertigo symptoms compared to alternative cochleostomy methods [38,47]. Electrode insertion depth shows no statistically significant correlation with subjective dizziness or observed nystagmus [36,37]. Various vestibular tests such as caloric testing, cVEMPs, and SVV confirm this finding, although methodological differences and small sample sizes limit comparability. The sensitivity of individual vestibular tests is generally low, and subjective dizziness correlates only weakly with objective vestibular test results after CI, which restricts the informative value of single-modality investigations and supports multimodal assessment [6]. Limitations such as retrospective study design, different imaging methods for electrode position control, and variable follow-up periods complicate interpretation.

Besides the small study cohort, a limitation in interpreting the findings arises from generally elevated pre- and postoperative vHIT gain values (>1) and high standard deviations. The relatively wide age range of the study population (22–90 years) reflects routine clinical practice and may have contributed to the variability of vestibular measurements. Age-related differences in vestibular function may therefore have contributed to the observed variability and should be considered when interpreting the vHIT results. Age was not included as a covariate. The primary comparison is a within-subject paired preoperative-to-postoperative comparison, in which age is constant within each individual and therefore cannot confound the estimated change. Moreover, large normative datasets acquired with the same device report that horizontal VOR gain is largely preserved across decades into the ninth decade of life [32], while other work has described a small age-associated decrease [48]. An age effect of this magnitude is unlikely to account for the differences observed here, although age may contribute to the between-subject variability of absolute gain values.

Several factors might contribute to these findings, including loose-fitting video goggles causing “goggle slippage”, which can artificially increase vHIT gain [16]. The vHIT measurements were performed using the EyeSeeCam™ system, which calculates gain based on high-frequency head impulses. Elevated standard deviations may reflect variability in head impulse execution, patient compliance, or residual artefacts despite quality control measures. Additionally, anxiety and arousal levels may play a role, as increased physiological arousal has been associated with elevated vHIT gain [49]. Other contributing factors include head-hand positioning, target distance reduction, and camera alignment [50,51,52]. These methodological factors may also have influenced the observed differences between pre- and postoperative examinations. Consequently, the reduction in vHIT gain observed in the left-sided subgroup should be interpreted with caution. Given the limited sample size and the lack of correction for multiple comparisons, this finding should be considered exploratory and cannot be considered definitive evidence of postoperative vestibular dysfunction. Rather, the present findings indicate a possible subtle postoperative alteration that requires confirmation in prospective studies using standardized testing conditions. Given these confounding variables, the occurrence of catch-up saccades should be interpreted in conjunction with vHIT gain measurements, as accurate assessment of vestibular function requires both parameters and careful minimization of methodological confounders [15].

A further limitation is that catch-up saccades were assessed qualitatively rather than quantitatively. Although saccades were classified as present or absent by a blinded experienced evaluator, quantitative parameters such as saccade amplitude, latency, or covert versus overt distribution were not systematically analyzed. Given the limited sample size and the resulting small subgroups, further subdivision according to saccade characteristics would have substantially reduced the statistical interpretability of the analyses. Future prospective studies with larger cohorts should therefore include quantitative assessment of catch-up saccades and longitudinal follow-up to determine their clinical relevance and persistence over time.

Another limitation of this study is the lack of standardized assessment of subjective dizziness, which restricts the interpretation of symptom–function relationships.

Additionally, the potential role of preoperative vestibular rehabilitation warrants further investigation in prospective studies, particularly in patients considered at increased risk for postoperative vestibular symptoms. Furthermore, future longitudinal studies using standardized vestibular assessments should investigate vestibular outcomes after bilateral CI.

In the long term, multicenter studies with standardized, multimodal protocols including comprehensive vestibular diagnostics are recommended to gain more reliable insights and develop practical postoperative concepts. Future studies should consider assessing additional vestibular structures, including the vertical semicircular canals, particularly the posterior semicircular canal, as well as otolith function using oVEMP and cVEMP. Such a multimodal approach may provide a more comprehensive assessment of vestibular changes following CI.

The current data reflect clinical reality but do not yet allow definitive conclusions about the relationship between subjective dizziness and objective vestibular function.

Taken together, the present findings may be summarized at three levels. First, what the study demonstrates: In this retrospective cohort, the prespecified primary comparison of the implanted ear detected no statistically significant change after right-sided CI, and no significant change was detected in SVV or between the implanted and the non-implanted ear. Because a non-significant result is not evidence of equivalence, and because no equivalence or non-inferiority margin was defined, these findings must not be read as demonstrating that vestibular function was preserved; the confidence interval of the right-sided comparison remains compatible with a reduction in gain of up to approximately 0.41 units, and for the left-sided comparison, where a significant reduction was found, no interval estimate is available at all. Second, what the study suggests: Exploratory within-group analyses showed a postoperative reduction in 60 ms vHIT gain in the left-sided subgroup and a higher frequency of left-directed catch-up saccades in the same subgroup, which may indicate subtle postoperative changes in horizontal vestibulo-ocular metrics. Third, what the study does not establish: It does not establish clinically meaningful vestibular hypofunction, a biological left-sided susceptibility, a causal relationship between implantation and the observed changes, or receptor-specific otolith damage. Because the available test battery comprised horizontal vHIT and SVV only, and caloric testing, cVEMP, oVEMP, and vertical-canal vHIT were not performed, no conclusion regarding saccular function or vestibular function as a whole after CI can be drawn from these data. It should also be emphasized that 38.9% of the participants with paired data developed new ipsilateral catch-up saccades, a change that reached statistical significance in the exact McNemar analysis for both implantation sides in the total cohort, and that this descriptive finding is difficult to reconcile with any claim of preserved horizontal vestibulo-ocular function.

Finally, several features limit the precision of the present estimates. With 43 patients overall and side-stratified subgroups of only 14 to 19 patients, the study is vulnerable both to false-negative findings due to limited power and to unstable effect estimates in small-cell comparisons; the direction-specific catch-up saccade analysis, in which one cell contained a single patient, is the clearest example. The sample size was determined by the availability of eligible paired clinical data rather than by an a priori power calculation, and retrospective observed-power calculations were deliberately omitted because they add no information beyond the reported *p*-values [31]. In addition, postoperative vHIT and SVV were obtained at systematically different intervals after surgery, and all patients with missing paired vHIT data had undergone right-sided implantation. The side-stratified results are therefore reported as hypothesis-generating and should be confirmed in adequately powered prospective cohorts reporting effect estimates with confidence intervals rather than significance tests alone. Two further limitations follow from the above. The postoperative assessment interval differed systematically between the two modalities and was not analyzed as a covariate, so vHIT and SVV findings must not be integrated as though they described a common postoperative state; because the direct comparison of individual change scores between implantation sides was not performed, the side-stratified pattern remains untested rather than merely unexplained.

## 5. Conclusions

In this retrospective cohort, the prespecified primary comparison of the implanted ear detected no statistically significant change after right-sided CI (mean paired change: +0.07; 95% CI: −0.41 to 0.56), whereas a reduction was found after left-sided CI (*p* = 0.003); the two sides were not compared formally. No significant change was detected in SVV. Given the width of the available interval and the small sample, these results do not demonstrate that vestibular function was preserved, and 38.9% of participants with paired data developed new ipsilateral catch-up saccades. No statistically significant association was detected between electrode insertion depth and vestibular function in this exploratory and methodologically limited analysis.

These observations represent an exploratory signal compatible with, but not specific to, subtle postoperative changes in the recorded vestibulo-ocular metrics; their clinical significance and reproducibility require confirmation in larger prospective studies using standardized longitudinal and multimodal vestibular assessment.

## Figures and Tables

**Table 1:** Paired preoperative and postoperative ipsilateral catch-up saccade: Classification of catch-up saccades on the implanted (ipsilateral) side according to preoperative and postoperative vHIT findings. The total cohort comprised 43 patients; paired data were available for 17 patients with right-sided CI and 19 patients with left-sided CI. The subgroup without prior ear surgery comprised 16 patients with right-sided CI and 14 with left-sided CI with paired data.

CI Side	Paired Patients (n)	Absent → Absent Unchanged	Absent → Present New Postoperative	Present → Absent Resolved	Present → Present Persistent	McNemar *p*-Value
Right-sided CI (total cohort)	17	9 (52.9%)	6 (35.3%)	0 (0.0%)	2 (11.8%)	0.031
Left-sided CI (total cohort)	19	8 (42.1%)	8 (42.1%)	1 (5.3%)	2 (10.5%)	0.039
Right-sided CI (no prior ear surgery)	16	9 (56.3%)	5 (31.3%)	0 (0.0%)	2 (12.5%)	0.063
Left-sided CI (no prior ear surgery)	14	5 (35.7%)	7 (50.0%)	1 (7.1%)	1 (7.1%)	0.070

## Data Availability

The data that support the findings of this study are not publicly available because they contain information that could compromise the privacy of research participants but are available from the corresponding author [LZ] upon request.
